# Studies on Antimicrobial and Immunomodulatory Effects of Hot Aqueous Extract of *Acacia nilotica* L. Leaves against Common Veterinary Pathogens

**DOI:** 10.1155/2014/747042

**Published:** 2014-04-07

**Authors:** Arvind Kumar Sharma, Amit Kumar, Sharad Kumar Yadav, Anu Rahal

**Affiliations:** ^1^Department of Veterinary Microbiology, College of Veterinary Sciences and Animal Husbandry, Uttar Pradesh Pandit Deen Dayal Upadhyaya Pashu Chikitsa Vigyan Vishwavidyalaya Evam Go Anusandhan Sansthan (DUVASU), Mathura 281001, India; ^2^Department of Veterinary Pharmacology and Toxicology, College of Veterinary Sciences and Animal Husbandry, Uttar Pradesh Pandit Deen Dayal Upadhyaya Pashu Chikitsa Vigyan Vishwavidyalaya Evam Go Anusandhan Sansthan (DUVASU), Mathura 281001, India

## Abstract

*Acacia nilotica* is a plant species that is almost ubiquitously found in different parts of the world. Various preparations of it have been advocated in folk medicine for the treatment of tuberculosis, leprosy, smallpox, dysentery, cough, ophthalmia, toothache, skin cancer as astringent, antispasmodic, and aphrodisiac since immemorial times. The present study investigates the antibacterial, antifungal, antiviral, and immunomodulatory potential of hot aqueous extract (HAE) of *Acacia nilotica* leaves. On dry matter basis, the filtered HAE had a good extraction ratio (33.46%) and was found to have carbohydrates, glycosides, phytosterols, phenolic compounds, saponins, and flavonoids as major constituents. HAE produced dose dependent zone of inhibition against *Klebsiella pneumoniae, Pseudomonas aeruginosa, E. coli, Bacillus cereus, Staphylococcus aureus, and Streptococcus uberis* and fungal pathogens *Aspergillus niger* and *Aspergillus fumigates*; however, no antiviral activity was recorded against IBR virus. HAE of *A. nilotica* revealed both proliferative and inhibitory effects on the rat splenocytes and IL-10 release depending on the dose. Detailed studies involving wide spectrum of bacterial, fungal, and viral species are required to prove or know the exact status of each constituents of the plant extract.

## 1. Introduction

The genus* Acacia* is the second largest in the family Leguminosae, with about 1350 species. It is distributed throughout tropical and warm temperate areas of the world, with the largest concentration of species in Australia (957 species), The Americas (185 species), Africa (144 species), and Asia (89 species) [1]. Out of these,* Acacia nilotica* is one of the species that has been effectively utilized in folk medicine for the treatment of tuberculosis, leprosy, smallpox, dysentery, cough, ophthalmia, toothache, skin cancer as astringent, antispasmodic, and aphrodisiac by rural population [2, 3].* Acacia nilotica* leaves are protein rich and highly digestible. Leaves of Acacia plants, in general, possess a significant level of antibacterial activity against a wide range of bacterial pathogens, although the extent of antibacterial activity varies depending upon the type of extract [4–8]. Various extracts in water, methanol, ethanol, n-hexane, Chloroform, and petroleum ether are reported with variable antibacterial activity against Gram-positive and Gram-negative bacteria [7–12], common fungal pathogens [13–17], and viruses [18–21]. Different preparations of* A. nilotica* leave have been reported with different phytoconstituents [7, 22, 23]. These constituents of extracts also revealed variable potential for blood cells proliferation [24] and immunomodulation [25–27]. However, a complete composite study on all the potential activities of aqueous extract of* A. nilotica*, the most commonly used extract in households, is lacking against common yet potential veterinary pathogens. Thus, the present study was planned to evaluate common phytochemical constituents of aqueous extract with antimicrobial activity against common pathogens of veterinary importance and its role in splenocyte stimulation to influence immune response and cytokine induction.

## 2. Materials and Methods

### 2.1. Collection and Processing of* A. nilotica* Leaves

The authenticated and verified plant leaves were included in the study. Procured leaves were washed with single glass distilled water and dried at 37°C in incubator. The dried plants samples were ground to prepare a coarse powder. The powdered samples were used for preparation of HAE using soxhlet apparatus [23].

#### 2.1.1. Extracts Preparation

Triple glass distilled water was used as solvent for HAE preparation using Soxhlet apparatus as per previously described method [28].

#### 2.1.2. Determination of Yields of Extract

The evaporated dried extracts (on dry weight basis) were calculated by the following equation [29]:

(1) $$\begin{array}{c} \text{Yield}\;\left(\frac{\text{g}}{100\;\text{g}\;\text{of}\;\text{dry}\;\text{plant}\;\text{material}}\right)=\frac{\left(W_{1}\times 100\right)}{W_{2}}, \end{array}$$

where *W*_1_ was the weight of the extract after the solvent evaporation and *W*_2_ was the weight of the dry plant material loaded for extraction.

#### 2.1.3. Phytochemical Studies

Qualitative phytochemical analysis of crude hot aqueous extract was carried out as per the standard methods [30, 31] to detect the presence or absence of different phytochemical constituents, namely, alkaloids, glycosides, flavonoids, resins, tannins, saponins, fixed oils, reducing sugars, proteins, and amino acids.

#### 2.1.4. Disc Preparation

Blank sterile discs (Himedia, Mumbai, India) of 6 mm diameter were loaded with HAE of different concentrations (1000 mg/mL, 500 mg/mL and 250 mg/mL) to prepare discs containing 20 mg, 10 mg, 5 mg, 2.5 mg, and 1.25 mg extracts. Discs impregnated with sterilized triple distilled water were taken as negative control. Discs were allowed to dry at 40°C for 30 minutes [23]. Dried discs were tested for their sterility on nutrient and Sabouraud Dextrose agar (SDA).

### 2.2. Antimicrobial Activities

#### 2.2.1. Bacterial, Fungal, and Virus Isolates

Bacterial cultures of* Staphylococcus aureus, Bacillus cereus, Streptococcus uberis, Escherichia coli, Pseudomonas aeruginosa*, and* Klebsiella pneumonia* and fungal cultures of* Candida albicans, Aspergillus niger,* and* Aspergillus fumigatus* were obtained from the Department of Microbiology and Immunology, UP Pt. Deen Dayal Upadhyaya Pashu Chikitsa Vigyan Vishwavidyalaya Evam Go Anusandhan Sansthan (DUVASU), Mathura, to determine* in vitro* antibacterial and antifungal activity of HAE. Prior to use, the bacterial and fungal isolates were recharacterized on the basis of morphological, cultural, and biochemical characteristics as per standard methods [32]. For antiviral activity, Infectious Bovine Rhinotracheitis (IBR) virus was obtained from the Department of Epidemiology, UP Pt. Deen Dayal Upadhyaya Pashu Chikitsa Vigyan Vishwavidyalaya Evam Go Anusandhan Sansthan, (DUVASU), Mathura. Before its use, IBR virus was subcultured and characterized by cytopathic effect in Madin-Darby Bovine Kidney (MDBK) cell line.

#### 2.2.2. *In Vitro* Study of Antibacterial Susceptibility

Bacterial cell concentration of the bacterial cultures was determined on Muller Hinton Agar [HiMedia, Mumbai] by Spread Plate Method and colony forming unit per mL (CFU/mL) were calculated by multiplying the number of colonies counted with respective dilution factor [33]. Antibacterial activity was carried out by disc diffusion method as per standard procedure [34]. Standard discs of antibiotic Tetracycline (30 *μ*g) and Amikacin (30 *μ*g) [Hi Media, Mumbai] were used as positive control for Gram-positive and Gram-negative bacteria, respectively.

#### 2.2.3. *In Vitro* Antifungal Effect

The concentration of CFU of fungus was determined as per the method used for bacterial count except for the use of Sabouraud's Dextrose Agar (SDA) [HiMedia, Mumbai] in place of Muller Hinton Agar.* In vitro* study of antifungal susceptibility of plant extract was performed by standard disc diffusion method [34]. Standard antimycotic discs of Fluconazole (10 *μ*g) [Hi Media, Mumbai] were used as positive control for fungal agents.

#### 2.2.4. *In Vitro* Antiviral Effect against IBR Virus

MDBK cell lines were obtained from the Department of Epidemiology, UP Pt. Deen Dayal Upadhyaya Pashu Chikitsa Vigyan Vishwavidyalaya Evam Go Anusandhan Sansthan, (DUVASU) Mathura. These were maintained at 37°C, 5% CO_2_, and 80% relative humidity using Dulbecco's Modified Eagle's Medium (DMEM) growth medium supplemented with 10% inactivated fetal calf serum, antibiotic antimycotic solution, sodium pyruvate, and sodium bicarbonate [35] to study the antiviral effect of HAE. To assess the antiviral effect the cellular toxicity of plant extract on MDBK cell line was carried by MTT {3-(4,5-Dimethylthiazolyl)-2,5-diphenyl tetrazolium bromide} dye method [36] in 96-well cell culture plates. The concentration at which there was no reduction of viable cells was considered as maximum nontoxic dose (MNTD) of the extract [37]. The concentrations of aqueous extract lower than MNTD were screened for antiviral property against TCID_50_ virus challenge dose of IBR virus in MDBK cell lines. The antiviral effect was determined by cytopathic inhibition effect in MDBK cell lines and reduction in virus induced cytotoxicity was measured by using MTT dye uptake method [36] in the form of optical densities (O.D) [37]. The virus suspension and dilution medium without plant extract were also used as the virus control and cell control, respectively. Cell viability was evaluated by adding 10 *μ*L MTT dye (5 mg/mL) and then extracting the dye with DMSO and measuring optical density (O.D.) at 560 nm–670 nm as described previously. The percentage protection was calculated by the following formula:

(2) $$\begin{array}{c} \frac{\left(\text{ODt}\right)v-\left(\text{ODc}\right)v}{\left(\text{ODc}\right)m-\left(\text{ODc}\right)v}\times 100, \end{array}$$

where (ODt)*v*, (ODc)*v*, and (ODc)*m* correspond to absorbance in virus infected cells with plant extracts, virus infected cells without plant extracts, and cells without virus and plant extract, respectively [35, 37].

### 2.3. Immunomodulatory Activities

#### 2.3.1. Experimental Animals

Wistar Albino Rats (Av wt-120 gm) were procured from the Department of Lab Animal Resource (LAR) of IVRI, Izatnagar, UP. The spleen cells were collected aseptically from the rats and used for spleenocyte proliferation and cytokine IL-10 analysis [35].

#### 2.3.2. *In Vitro* Effect on Splenocytes Proliferation/Inhibition

The effect of HAE on splenocyte proliferation was evaluated with wells containing only spleen cells as negative control and wells containing spleen cells with Con-A as positive control [35]. After incubation, 20 *μ*L of MTT solution (5 mg/mL) was added in each well for formazione crystal formation. The plate was reincubated at 37°C for 4 hrs in CO_2_ incubator containing 5% CO_2_ and 80% relative humidity. After incubation, supernatant was removed. The plate was air dried and 100 *μ*L of DMSO was added to dissolve the formazione crystals. O.D was taken at dual wavelength 560–670 nm by ELISA reader [35]. Mean values were calculated in comparison to control and taken positive if ratio was of significant difference [38].

## 3. *In Vitro* Effect on IL-10 Cytokine Induction

Splenocytes were prepared as prescribed for splenocyte proliferation assay and cultured in absence and presence of Con-A (5 mg/mL) along with 0.45 *μ*m membrane filtered HAE of plants (31.25, 62.5, 125, 250, and 500 *μ*g/mL, extract) in the cell culture plate [39]. Procedure followed for IL-10 assay was similar to that for splenocytes proliferation assay. However, the spleen cells were incubated for 48  hrs and then supernatant was collected for the detection of IL-10 cytokine. The quantitation of IL-10 cytokine in spleen culture supernatant was done according to the protocol supplied in the kit by BIOSOURCE (USA).

## 4. Results

### 4.1. Percentage Yield

On dry matter basis, the filtered HAE of* Acacia nilotica* leaves was 33.46% of the total dry weight of the leaves.

### 4.2. Phytochemical Studies

Phytochemical studies of HAE revealed the presence of carbohydrates, glycosides, phytosterols, phenolic compounds, saponins, and flavonoids as major constituents.

### 4.3. *In Vitro* Antibacterial Effects

All the bacterial pathogens revealed concentration dependent sensitivity against HAE. HAE was more effective against Gram-positive bacteria in comparison to Gram-negative bacteria (Table 1).

### 4.4. *In Vitro* Antifungal Effects

All the fungal pathogens revealed concentration dependant sensitivity against higher concentrations of HAE. The discs with 2.5 and 5 mg concentration of HAE revealed no inhibition or the inhibition of the growth for initial few hours (Table 2).

### 4.5. *In Vitro* Antiviral Activity against IBR Virus 

#### 4.5.1. MNTD Determination in MDBK Cell Line

**Figure 1:**
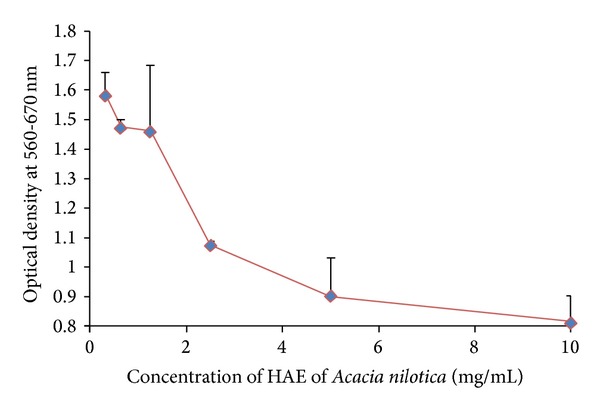
MNTD of HAE of* Acacia nilotica leaves* in MDBK cell line.

On the basis of observation of cytotoxic effect (vacuole formation and detachment of cells) in MDBK cells and absorbance, 1.25 mg/mL conc. of aqueous extract was determined as nontoxic dose of HAE. For further investigation, 1.25, 0.625, and 0.3125 mg/mL were taken to study the antiviral activity of HAE of* A. nilotica* leaves against IBR virus (Figures 1 and 6).

#### 4.5.2. TCID_50_ for IBR Virus

**Figure 2:**
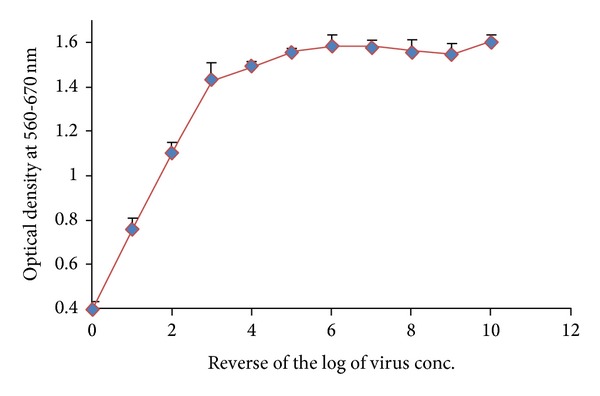
TCID_50_ for Infectious Bovine Rhinotracheitis (IBR) virus.

The number of viable cells after virus challenge was assessed by using MTT dye uptake assay and the virus dilution required to cause 50% cell death was calculated from dose response curve and virus was expressed as TCID_50_ (50% tissue culture infective dose). TCID_50_ of IBR virus was determined at 10^−1^ virus dilution level (Figure 2).

#### 4.5.3. Antiviral Effect against IBR Virus

**Figure 3:**
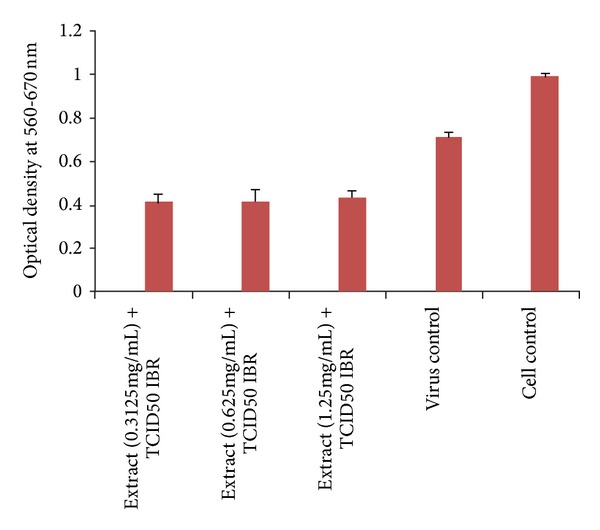
Antiviral effect of HAE of* Acacia nilotica* leaves against IBR virus.

HAE of* A. nilotica* leaves showed no antiviral property against IBR, showing no protection to the cell line against the virus (Figure 3).

### 4.6. *In Vitro* Effect on Splenocytes Proliferation/Inhibition

**Figure 4:**
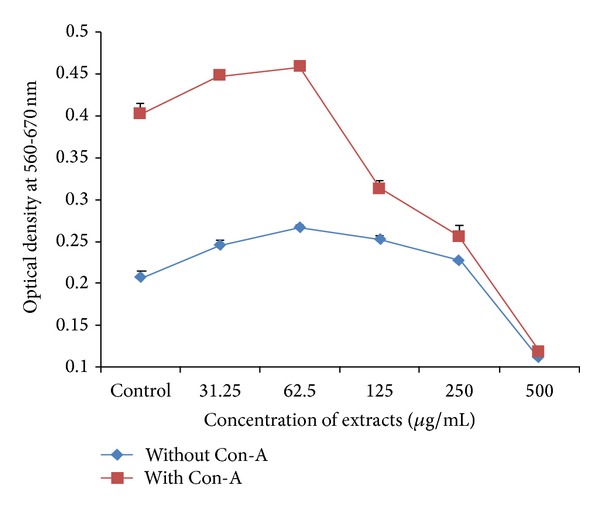
*In vitro* effect of HAE of* Acacia nilotica* leaves on Splenocyte proliferation in Wistar albino rats.

HAE of* A. nilotica* leaves had a proliferative as well as inhibitory effect on splenocytes. In comparison to negative control, 18.27, 28.36, 21.63, and 9.61% increase in the proliferation of spleen cells were reported at the dose rate of 31.25, 62.5, 125, and 250 *μ*g/mL of HAE, respectively, whereas 45.67% inhibition was reported at dose rate of 500 *μ*g/mL HAE of* A. nilotica*. In comparison to positive control, spleen cells with HAE of* Acacia nilotica* leaves in presence of Con-A exhibited 11.17% and 13.18% increase in proliferation when splenocyte culture was treated with 31.25 and 62.5 *μ*g/mL of HAE of* A. nilotica*, respectively. However, 22.33, 36.72, and 70.22% inhibition were observed when splenocyte culture treated with 125, 250, and 500 *μ*g/mL HAE of* A. nilotica* with Con-A, respectively (Figures 4 and 7).

### 4.7. *In Vitro* Effect on Induction of IL-10 Cytokine

**Figure 5:**
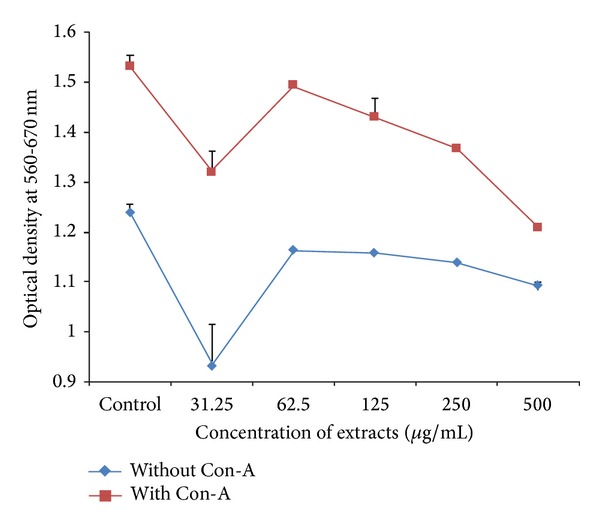
*In vitro* effect of HAE of* Acacia nilotica* leaves on Cytokine IL-10 induction.

**Figure 6:**
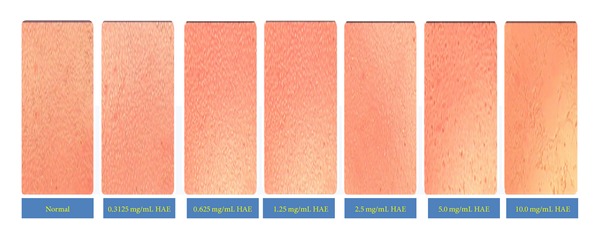
MNTD assessment with different concentrations of HAE of* A. nilotica* leaves in MDBK cell line, 10X.

Splenocytes treated with HAE* in vitro* revealed 24.78, 6.21, 6.69, 8.15, and 11.86% reduction in the IL-10 secretion at dose rate of 31.25, 62.5, 125, 250, and 500 *μ*g/mL HAE of* A. nilotica* leaves, respectively, compared with negative control (only spleen cells). The minimum inhibition of cytokine secretion (6.21%) was observed with 62.5 *μ*g/mL HAE in absence of Con-A (Figure 5).

Splenocytes treated with HAE and Con-A was compared with positive control (spleen cells + Con-A) and it was found that IL-10 secretion was reduced by 13.85, 2.42, 6.59, 10.78, and 21.09% when treated with 31.25, 62.5, 125, 250, and 500 *μ*g/mL HAE, respectively. The minimum reduction 2.42% was observed with 62.5 *μ*g/mL of HAE in presence of Con-A (Figure 5).

## 5. Discussion

*A. nilotica* is commonly located in Indian subcontinent [1] and is commonly used in folk medicine [40]. In traditional medicine, its role is well established [41]. However, in the era of increasing drug resistance to modern medicine in bacterial pathogens [42–44], it is to be validated scientifically. Out of the numerous traditional therapeutic practices, the preparation of hot aqueous extract is the most common household means of herbal administration [33, 40]. Therefore, the antimicrobial potential along with immunomodulatory effects of hot aqueous extract of* A. nilotica* leaves was investigated in the present study.

The high yield of the plant leaves extract (33.46%) also supports its use and availability in folk medicine. The phytochemicals like tannins, flavonoids, phenols, and alkaloids are an important reflection of the pharmacological activities of a plant. The phytochemistry of the extract revealed the presence of carbohydrates, glycosides, phytosterols, phenolic compounds, saponins, and flavonoids as major constituents; the efficacy of these compounds is already well established for antimicrobial activities [8]. These findings are in agreement with earlier findings of phytoconstituents of different extracts of* Acacia nilotica,* namely, aqueous extract [22], methanolic, and ethanolic extracts [7, 23].

The HAE produced dose dependent zone of inhibition of variable size even after the incubation of 48 hours against both Gram-positive and Gram-negative bacteria (Table 1). These findings are in the concurrence with the earlier findings that reported better efficacy of* Acacia nilotica* extracts against Gram-positive cocci than Gram-negative bacilli [8]. The superior inhibition of bacterial growth against Gram-positive bacteria in comparison to Gram-negative bacteria might be because of the difference in cell wall composition of the bacteria [42]. Among Gram-negative bacteria,* Klebsiella pneumoniae* showed a little resistance to inhibition, perhaps due to the presence of a capsule [22]. The sensitivity of Gram-positive bacteria was in support to the use of HAE extracts in skin ailments and conditions like mastitis [45]. Similar to our findings,* E. coli* has been earlier found sensitive to hot aqueous, ethanolic, and methanolic extracts of* Acacia nilotica* [8, 9, 12, 14]. However, the alcoholic extracts are reported to have a better antibacterial activity against different bacterial pathogens with significant inhibition of growth as compared to the aqueous extracts [10, 15].

Other than the bacterial pathogens, mycotic pathogens are always a clinical challenge to veterinarian and are very difficult to control [46]. The fungal manifestations are generally chronic in nature. Although an exhaustive range of antifungal drugs are available, the treatment becomes uneconomical particularly in the large animals due to large dose size. Therefore, to find out a suitable plant extract with antifungal activity was one of the key aims of the study. In this regard, HAE of* Acacia nilotica* plant leaves was tested against most common veterinary fungal pathogens, namely,* Candida albicans, Aspergillus fumigatus,* and* Aspergillus niger* (Table 2). The HAE showed good antifungal activity against all the fungal pathogens at higher concentration (Table 2). The inhibition was better against* Aspergillus fumigatus* and* Aspergillus niger* and was comparable to fluconazole, the reference antifungal drug. Our findings are also in agreement to the earlier findings against eight species of Aspergillus and other fungal pathogens [15–17, 47]. In contrast to our findings, some earlier reports have claimed absence of antifungal activity in HAE and other extracts of these plant parts [13, 14, 48]. This dissimilarity might be due to the difference in the collection season of plant material, extraction procedures, or geographical variations. The activity of HAE of* A. nilotica* leaves against* C. albicans* was only with higher concentration and only for 24 hours, while ethanolic extract of* A. nilotica* leaves were reported to be very effective [11]. This variation is commonly observed with the change of type of extract as phytoconstituents and their concentrations may vary significantly [8].

The severity of viral diseases and ability of virus to survive intracellularly pose a great challenge that is further aggravated by the nonavailability of specific antiviral chemical agents against veterinary pathogens [37]. Thus, the evaluation of antiviral effect of HAE of* A. nilotica* leaves was also attempted. The findings of the present study revealed no antiviral effect of HAE against IBR virus (Figure 3). There are no reports available on the antiviral activity of HAE of* A. nilotica* against IBR virus in the literature. However, variable antiviral activity of different extracts of different species of plant acacia have been reported, namely,* Acacia nilotica* (bark and pods) inhibitory effects against HIV-1 PR. [18];* Acacia gummifera* inhibiting Sindbis virus [19];* Acacia arabica*. var.* indica* against peste des petits ruminants virus (PPRV) [49];* Acacia arabica* (babul) against Goat pox virus (GTPV) replication [21]; and* Acacia nilotica* (fruits) with mild virucidal to high activity against replication of Newcastle Disease virus and Fowl pox viruses [19, 21].

**Figure 7:**
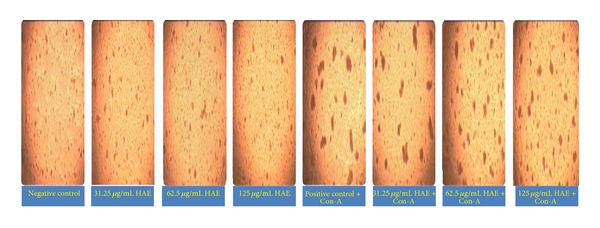
*In vitro* assessment of splenocyte proliferation with different concentrations of HAE of* A. nilotica* leaves on Wistar albino rat spleen cells, 10x.

HAE of plant leaves revealed both proliferative and inhibitory effects on the splenocytes to be depending on the concentration of the extract (Figures 4 and 7). The proliferation of splenocyte occurred in dose dependent manner in the range of 9.61% to the 28.36%. These observations are in agreement with the* in vitro* findings of stimulation of rat pleural polymorphonuclear leukocytes (PMNs) [24] and proliferation of splenocyte [26]. Thus, the lower concentrations of the HAE of plants could be used to improve immune response or to combat the microorganisms causing immune-suppression as increase in the proliferation of splenocyte appeared to be an indicative of cellular immune response [50]. Moreover, oral feeding of* Acacia catechu* extract in mice produced a significant increase in the serum immunoglobulin levels, increase in the haemagglutination titre values, and decreased the mortality ratio in mice, suggesting its effect on the humoral arm of the immune system [27]. The inhibition of splenocytes proliferation observed at higher concentration of HAE (Figures 4 and 7) might be due to the accumulation of toxic constituents of plant extract which might be causing cytotoxic effects and ultimately inhibiting the cell proliferation [26]. The supernatants of splenocytes exposed to different concentrations of extract were assessed for the concentration of IL-10, an anti-inflammatory interleukin that plays important role in control of inflammatory process [40, 51]. The HAE of* Acacia nilotica* downregulated the IL-10 release from spleen cells in lower doses and maximum inhibition was 24.78%. Then inhibition was reduced with increase in HAE concentration, however further higher doses downregulated IL-10 release. Hence, dose dependent upregulation is observed in certain concentration which is again downregulated with increase in extract concentration (Figure 5). Thus, proliferation of splenocytes and upregulation of IL-10 can be observed for an optimum concentration of extract. No literature is available on IL-10 regulation regarding this plant. Further, there are scanty reports available on the use of medicinal plants or the extracts of plant parts on the regulation of IL-10 as Fu-ling Chinese herb produced upregulation of IL-10 in murine spleen cell [52] and* Phyllanthus amarus* inhibited induction of interleukin (IL)-1*β*, IL-10, and interferon-*γ* in human whole blood [53]. Since cytokines regulate certain important biological functions such as cell growth, cell activation, immunity, inflammation, tissue repair, fibrosis, morphogenesis, and chemotaxis, thus the up- or downregulation of cytokines directly affect the body defense mechanism.

## 6. Conclusions

The study had tried to cover almost all the aspects of microbial pathogenesis and revealed that HAE of* Acacia nilotica* leaves had different effects upon bacteria, virus, and fungi with dose dependent variation in immunomodulatory and anti-inflammatory activities. HAE of* Acacia nilotica* leaves showed excellent antibacterial and antifungal activities with comparatively lesser antiviral and immunomodulatory activities. HAE of* Acacia nilotica* leaves had various active components that might have a role in variation in overall activities; therefore, further detailed studies involving wide spectrum of bacterial, fungal, and viral species are required to prove the exact statics of each constituent of the plant extract. However, study had shown an important level of the valuable effects of* Acacia nilotica* leaves.

## Supplementary Material

The supplementary files are available in the form of tables depicting the experimental schedule for *In-vitro* Splenocytes proliferation; IL-10 cytokine analysis, ELISA Test Protocol and OD values for MNTD estimation of HAE of *Acacia nilotica* leaves in MDBK cell line, titration for TCID_50_ for IBR virus, antiviral effect of HAE of Acacia nilotica leaves against IBR virus, *In-vitro* effect of HAE of *Acacia nilotica* leaves on Splenocyte proliferation in Wistar albino rats and *In-vitro* effect of HAE of *Acacia nilotica* leaves on Cytokine IL-10 induction.

## Figures and Tables

**Table 1:** *In vitro* antibacterial effects.

S. no.	Name of bacteria	Quantity of extract (mg/disc)	Zone of inhibition (mm) after		
			24 hrs	36 hrs	48 hrs
1	*Staphylococcus aureus *	1.25	9	8	8
		2.5	10	9	9
		5	17	15	15
		10	20	19	19
		20	20	20	20
	Positive control	Tetracycline (30 µg)	30	30	30

2	*Bacillus cereus *	1.25	8	8	7
		2.5	12	10	9
		5	15	15	14
		10	18	17	17
		20	19	19	18
	Positive control	Tetracycline (30 µg)	23	23	23

3	*Streptococcus uberis *	1.25	9	9	9
		2.5	12	12	12
		5	16	16	16
		10	19	19	18
		20	22	22	22
	Positive control	Tetracycline (30 µg)	27	27	27

4	*Escherichia coli *	1.25	0	0	0
		2.5	8	8	7
		5	9	8	8
		10	11	10	10
		20	15	14	14
	Positive control	Amikacin (30 µg)	24	24	24

5	*Pseudomonas aeruginosa *	1.25	8	8	7
		2.5	10	9	8
		5	13	12	12
		10	15	13	13
		20	15	13	12
	Positive control	Amikacin (30 µg)	18	18	18

6	*Klebsiella pneumoniae *	1.25	9	9	8
		2.5	10	9	8
		5	13	12	12
		10	13	13	13
		20	13	13	13
	Positive control	Amikacin (30 µg)	25	25	25

7	Negative control	0	—	—	—

**Table 2:** *In vitro* antifungal effects.

S. no.	Name of fungus	Quantity of extract (mg/disc)	Zone of inhibition (mm) after		
			24 hrs	36 hrs	48 hrs
1	*Candida albicans *	2.5	—	—	—
		5	—	—	—
		10	10	—	—
		20	11	—	—
		Fluconazole (10 µg)	28	28	28

2	*Aspergillus niger *	2.5	—	—	—
		5	8	—	—
		10	8	8	7
		20	12	11	11
		Fluconazole (10 µg)	21	21	21

3	*Aspergillus fumigatus *	2.5	—	—	—
		5	8	—	—
		10	9	8	8
		20	13	13	12
		Fluconazole (10 µg)	23	23	23

4	Negative control	0	—	—	—
