# Supplementary material for: Studies on Antimicrobial and Immunomodulatory Effects of Hot Aqueous Extract of Acacia nilotica L. Leaves against Common Veterinary Pathogens
Source: Vet Med Int. 2014 Apr 7;2014:747042. doi: 10.1155/2014/747042 (PMC3996978; doi:10.1155/2014/747042)
Supplement: Supplementary file 1 — The supplementary files are available in the form of tables depicting the experimental schedule for In-vitro Splenocytes proliferation; IL-10 cytokine analysis, ELISA Test Protocol and OD values for MNTD estimation of HAE of Acacia nilotica leaves in MDBK cell line, titration for TCID50 for IBR virus, antiviral effect of HAE of Acacia nilotica leaves against IBR virus, In-vitro effect of HAE of Acacia nilotica leaves on Splenocyte proliferation in Wistar albino rats and In-vitro effect of HAE of Acacia nilotica leaves on Cytokine IL-10 induction. [file 747042.f1.pdf]

Table-1: Experimental schedule for *In-vitro* Splenocytes proliferation and IL-10 cytokine analysis.

Only spleen cells	Only spleen cells	Only spleen cells	Spleen cells + Con-A	Spleen cells + Con-A	Spleen cells + Con-A
Spleen cells + Extract 31.25µg/ml	Spleen cells + Extract 31.25µg/ml	Spleen cells + Extract 31.25µg/ml	Spleen cells + Extract 31.25µg/ml + Con-A	Spleen cells + Extract 31.25µg/ml + Con-A	Spleen cells + Extract 31.25µg/ml + Con-A
Spleen cells + Extract 62.5µg/ml	Spleen cells + Extract 62.5µg/ml	Spleen cells + Extract 62.5µg/ml	Spleen cells + Extract 62.5µg/ml + Con-A	Spleen cells + Extract 62.5µg/ml + Con-A	Spleen cells + Extract 62.5µg/ml + Con-A
Spleen cells + Extract 125µg/ml	Spleen cells + Extract 125µg/ml	Spleen cells + Extract 125µg/ml	Spleen cells + Extract 125µg/ml + Con-A	Spleen cells + Extract 125µg/ml + Con-A	Spleen cells + Extract 125µg/ml + Con-A
Spleen cells + Extract 250µg/ml	Spleen cells + Extract 250µg/ml	Spleen cells + Extract 250µg/ml	Spleen cells + Extract 250µg/ml + Con-A	Spleen cells + Extract 250µg/ml + Con-A	Spleen cells + Extract 250µg/ml + Con-A
Spleen cells + Extract 500µg/ml	Spleen cells + Extract 500µg/ml	Spleen cells + Extract 500µg/ml	Spleen cells + Extract 500µg/ml + Con-A	Spleen cells + Extract 500µg/ml + Con-A	Spleen cells + Extract 500µg/ml + Con-A

Table-2: ELISA Test Protocol

+ve control Sup.	+ve control Sup.	A1	A1	B1	B1	C1	C1	D1	D1	E1	E1
-ve control Sup.	-ve control Sup.	A2	A2	B2	B2	C2	C2	D2	D2	E2	E2
Std 1	Std 1	Std 2	Std 2	Std 3	Std 3	Std 4	Std 4	Std 5	Std 5	Std 6	Std 6
RB	RB										

+ve control Sup. = spleen cells supernatant + Con-A, -ve control Sup. = spleen cells supernatant only, RB = Reagent blank, A1-E1=500/250/125/62.5/31.25µg/ml extract and Con-A treated supernatant, respectively. A2-E2= 500/250/125/62.5/31.25µg /ml, extract treated supernatant, respectively. Std1-6= Rat IL-10 standard of concentration 1000/500/250/125/62.5/31.25 pg/ml, respectively.

***Table-3: In-vitro* antibacterial effects of HAE:**

S.No.	Name of Bacteria	Quantity of extract (mg/disc)	Zone of Inhibition (mm) after		
			24 hrs	36 hrs	48 hrs
1.	*Staph. aureus*	1.25	9	8	8
		2.5	10	9	9
		5	17	15	15
		10	20	19	19
		20	20	20	20
	Positive Control	Tetracycline (30µg)	30	30	30
2.	*Bacillus cerus*	1.25	8	8	7
		2.5	12	10	9
		5	15	15	14
		10	18	17	17
		20	19	19	18
	Positive Control	Tetracycline (30µg)	23	23	23
3.	*Streptococcus uberis*	1.25	9	9	9
		2.5	12	12	12
		5	16	16	16
		10	19	19	18
		20	22	22	22
	Positive Control	Tetracycline (30µg)	27	27	27
4.	*E. coli*	1.25	0	0	0
		2.5	8	8	7
		5	9	8	8
		10	11	10	10
		20	15	14	14
	Positive Control	Amikacin (30 µg)	24	24	24
5.	*Pseudomonas aeruginosa*	1.25	8	8	7
		2.5	10	9	8
		5	13	12	12
		10	15	13	13
		20	15	13	12
	Positive Control	Amikacin (30 µg)	18	18	18
6.	*Klebsiella pneumoniae*	1.25	9	9	8
		2.5	10	9	8
		5	13	12	12
		10	13	13	13
		20	13	13	13
	Positive Control	Amikacin (30 µg)	25	25	25
7.	Negative control	0	–	–	–

Table-4: ***In-vitro* antifungal effects of HAE:**

S.No.	Name of Fungus	Quantity of extract (mg/disc)	Zone of Inhibition (mm) after		
			24 hrs	36 hrs	48 hrs
1.	***Candida albicans***	2.5	–	–	–
		5	–	–	–
		10	10	–	–
		20	11	–	–
		Fluconazole (10µg)	28	28	28
2.	***Aspergillus niger***	2.5	–	–	–
		5	8	–	–
		10	8	8	7
		20	12	11	11
		Fluconazole (10µg)	21	21	21
3.	***Aspergillus fumigates***	2.5	–	–	–
		5	8	–	–
		10	9	8	8
		20	13	13	12
		Fluconazole (10µg)	23	23	23
4.	Negative control	0	–	–	–

Table-5: MNTD of HAE of *Acacia nilotica* leaves in MDBK cell line

S.No.	Conc. of HAE of *A. nilotica* (mg/ml)	Absorbance (O.D)* (Mean ± S.E.)	Control (Mean ± S.E.)
1.	0.3125	1.579 ± 0.083	1.190 ± 0.046
2.	0.625	1.471 ± 0.028	
3.	1.25	1.458 ± 0.228	
4.	2.5	1.076 ± 0.012	
5.	5	0.905 ± 0.125	
6.	10	0.812 ± 0.091	

Difference (O.D. at 560 - 670 nm)*

Table-06: $TCID_{50}$ for IBR virus

S.N.	Log of virus concentration	Optical density* (Mean ± S.E.)
1	0	0.400 ± 0.031
2	-1	0.757 ± 0.049
3	-2	1.102 ± 0.048
4	-3	1.437 ± 0.070
5	-4	1.491 ± 0.022
6	-5	1.561 ± 0.013
7	-6	1.586 ± 0.051
8	-7	1.587 ± 0.025
9	-8	1.561 ± 0.051
10	-9	1.553 ± 0.044
11	-10	1.61 ± 0.025
12	Control	1.546 ± 0.046
Difference (O.D. at 560 - 670 nm)*		

Table-07: Antiviral effect of HAE of *Acacia nilotica* leaves against IBR virus

S.N.		Absorbance (O.D.)* (Mean ± S.E.)		
		0.3125mg/ml	0.625mg/ml	1.25mg/ml
1	HAE of *A. nilotica* + $TCID_{50}$ IBR	0.413 ± 0.037	0.414 ± .052	0.433 ± .028
2	Virus control	0.710 ± 0.020		
3	Cell control	0.992 ± 0.013		
4.	Percentage protection	Not Detectable		

Difference (O.D. at 560 - 670 nm)*

Table-08: *In-vitro* effect of HAE of *Acacia nilotica* leaves on Splenocyte proliferation in Wistar albino rats

S.No.	Conc. of extract (µg/ml)	Optical densities* (Mean ± S.E.)		% Proliferation/inhibition	
		Without Con-A	With Con-A	Without Con-A	With Con-A
1	Control**	0.208 ± 0.006	0.403 ± 0.012		
2	31.25	0.246 ± 0.005	0.448 ± 0.004	18.27	11.17
3	62.5	0.267 ± 0.002	0.459 ± 0.004	28.36	13.18
4	125	0.253 ± 0.004	0.313 ± 0.009	21.63	-22.33
5	250	0.228 ± 0.001	0.255 ± 0.015	9.61	-36.72
6	500	0.113 ± 0.006	0.120 ± 0.003	-45.67	-70.22

Difference (O.D. at 560 - 670 nm)*

Control** without Con-A = -ve Control = only spleen cells.

Control** with Con-A = +ve Control = Spleen cells + Con-A.

Table-09: *In-vitro* effect of HAE of *Acacia nilotica* leaves on Cytokine IL-10 induction

S.No.	Conc. of extracts (µg/ml)	Optical densities* (Mean ± S.E.)		% Change	
		Without Con-A	With Con-A	Without Con-A	With Con-A
1	Control**	1.239 ± 0.014	1.531 ± 0.023		
2	31.25	0.932 ± 0.082	1.319 ± 0.042	-24.78	-13.85
3	62.5	1.162 ± 0.000	1.494 ± 0.000	-6.21	-2.42
4	125	1.156 ± 0.002	1.430 ± 0.036	-6.69	-6.59
5	250	1.138 ± 0.002	1.366 ± 0.000	-8.15	-10.78
6	500	1.092 ± 0.006	1.208 ± 0.001	-11.86	-21.09

Control** without Con-A = -ve Control = only spleen cells.

Control** with Con-A = +ve Control = Spleen cells + Con-A.

Optical densities* (absorbance at 450nm)
